# Combined use of steady-state fluorescence emission and anisotropy of merocyanine 540 to distinguish crystalline, gel, ripple, and liquid crystalline phases in dipalmitoylphosphatidylcholine bilayers

**DOI:** 10.1186/1757-5036-3-14

**Published:** 2010-11-05

**Authors:** Hannabeth A Franchino, Brett C Johnson, Steven K Neeley, Rajeev B Tajhya, Mai P Vu, Heather A Wilson-Ashworth, John D Bell

**Affiliations:** 1 Department of Physiology and Developmental Biology, Brigham Young University, Provo, Utah 84602, USA; 2 Department of Biology, Utah Valley State College, Orem, Utah 84058, USA

## Abstract

The various lamellar phases of dipalmitoylphosphadtidylcholine bilayers with and without cholesterol were used to assess the versatility of the fluorescent probe merocyanine 540 through simultaneous measurements of emission intensity, spectral shape, and steady-state anisotropy. Induction of the crystalline phase (L_c_') by pre-incubation at 4°C produced a wavelength dependence of anisotropy which was strong at 15 and 25°C, weak at 38°C, and minimal above the main transition (>~41.5°C) or after returning the temperature from 46 to 25°C. The profile of anisotropy values across this temperature range revealed the ability of the probe to detect crystalline, gel (L_β_'), and liquid crystalline (L_α_) phases. The temperature dependence of fluorescence intensity was additionally able to distinguish between the ripple (P_β_') and gel phases. In contrast, the shape of the emission spectrum, quantified as the ratio of merocyanine monomer and dimer peaks (585 and 621 nm), was primarily sensitive to the crystalline and gel phases because dimer fluorescence requires a highly-ordered environment. This requirement also explained the diminution of anisotropy wavelength dependence above 25°C. Repetition of experiments with vesicles containing cholesterol allowed creation of a phase map. Superimposition of data from the three simultaneous measurements provided details about the various phase regions in the map not discernible from any one of the three alone. The results were applied to assessment of calcium-induced membrane changes in living cells.

PACS Codes: 87.16.dt

## 1. Introduction

Fluorescence spectroscopy is a useful biological technique for studying membrane structure that can be applied directly to living cells. Measurements in real time with living cells, before and after treatments with pharmacological agents, are most easily accomplished using steady-state measurements. If one could increase the amount of biophysical information available from those measurements, it would reduce the need for dual labeling or comparisons of parallel experiments with different probes. For example, it has been found with the probe laurdan that measurement of both steady-state anisotropy and emission spectrum shape can yield more detailed information about membrane phase properties in both artificial and natural membranes than either measurement alone [1,2]. Merocyanine 540 (MC540) seems like an ideal candidate for a similar approach but with greater resolution and flexibility. Its emission intensity is environment-sensitive, which allows it to be used in flow cytometry experiments to quantify cell subpopulations with differing biophysical membrane properties [3,4]. It binds to the plasma membrane of most cells at low concentrations (*i.e. *Ref. [3-8]). Furthermore, it has been used extensively for studies of membrane properties during apoptosis (*i.e. *Ref. [3,4,8-12]). Because of the sensitivity of MC540 to lipid-packing density and the ability of the probe to partition into different membrane locations depending on lipid phase [7,13-17], we postulated that it might be able to provide detailed information about membrane dynamics through multiple simultaneous steady-state measurements. We have used bilayers made of dipalmitoylphosphatidylcholine (DPPC) with and without cholesterol to assess the extent to which such might be the case.

The characteristics of pure DPPC in its four lamellar phases have been well-studied with biophysical methods such as differential scanning calorimetry, x-ray diffraction, and various spectroscopic techniques[18-23]. These include solid-ordered phases-crystalline (L_c_'), gel (L_β_'), and ripple (P_β_')-and the liquid-disordered or liquid crystalline phase (L_α_). More recently, liquid-ordered (L_o_) phases have been observed in the presence of cholesterol [24-30]. Additionally, at specific cholesterol concentrations, the L_α _and L_o _phases become more complex due to formation of extended superlattice structures [31-33].

The L_c_' phase of pure DPPC is found at low temperatures and can be induced in vesicles when stored at -5°C for 2 hours or longer or at 4°C for more than 24 hours [18,34]. In this phase, both the hydrocarbon chains of the lipids as well as the hydrophilic heads are tightly packed with highly restricted motion [35]. An increase in temperature causes the lipids to undergo the sub-transition at ~18°C and enter the L_β_' phase, which is characterized by increases in the spatial area occupied by the phospholipids, rotational freedom of movement, and hydration and tilt of the heads away from the bilayer normal [35].

The transition between the L_β_' and P_β_' phases of pure DPPC is classified as the pre-transition (centered at about 33-34°C) and is usually detected with differential scanning calorimetry [20,21]. The P_β_' phase is characterized by an increase in the interfacial area of the membrane as the lipid polar head groups occupy greater space [35]. In the case of multilamellar vesicles, the surface morphology changes at this point from being planar to instead bending into a series of periodic ripples [35].

Merocyanine 540 has a negatively-charged sulfate group that creates a permanent dipole moment which affects the binding locations of the probe and its tendency to exist as either monomers or dimers in the membrane [36]. The monomeric form of MC540 resides near the lipid head groups at an orientation parallel to the phospholipid chains [16]. Alternatively, MC540 can form an anti-parallel "sandwich" dimer [17]. It has been proposed that this dimer occupies a different region of the membrane compared to the monomer, although the exact orientation of the probe and whether that region is deep or superficial in the membrane are uncertain [16,37]. Emission intensity spectra show that the monomer fluoresces at a shorter wavelength than the dimer (585 nm and 621 nm, respectively) [17,37]. While the overall dimer fluorescence intensity is lower than that of monomers, dimers do contribute a noticeable amount to the emission intensity of MC540 when they reside in a highly packed environment such as exists in the membrane below the main phase transition [17]. Dimers are known to be non-fluorescent in both aqueous environments and when occupying membranes in the liquid-crystalline phase [17]. The introduction of cholesterol to the membrane is also reported to decrease the fluorescence of the dimer [15].

In this study, we have examined the versatility of using simultaneous measurements of MC540 anisotropy, emission intensity, and spectral shape to resolve the various DPPC lamellar phases. These experiments were then applied to two-component bilayers with multiple mole fractions of cholesterol. Finally, the study was complemented with a brief proof-of-concept application to living cells.

## 2. Methods

### Formation of vesicles

Vesicles were made by combining 1 μmole DPPC (Avanti Polar Lipids, Birmingham, AL) dissolved in chloroform with 5-6.8 nmoles MC540 to create membranes with a 1:145-1:200 probe-to-lipid ratio. Control experiments at a variety of ratios demonstrated that MC540 caused minimal perturbation to the membrane structure at ratios less than 1:100. Samples were dried under N_2 _gas and re-suspended in 1 ml of pH 7 citrate buffer (20 mM sodium citrate/citric acid, 150 mM KCl). The co-dispersing of MC540 with lipids during vesicle preparation was done to ensure that the probe would be properly equilibrated with the bilayers. For some experiments, DPPC was mixed with cholesterol (both dissolved in chloroform) at various mole ratios (1 μmole total lipid) prior to drying and suspension in citrate buffer. Suspensions were then incubated in a shaking water bath at ≥ 50°C for 10-min intervals and vortexed between each incubation (total incubation time was one hour). The vesicles were stored in a covered container either at room temperature or at 4°C for at least 24 h before use. Samples containing cholesterol were kept refrigerated and used soon after preparation to minimize oxidation [38].

### Steady-state fluorescence measurements

Steady-state fluorescence was assayed using a Fluoromax-3 (Horiba Jobin Yvon, Edison, NJ) or PC-1 (ISS, Champaign, IL) photon-counting spectrofluorometer. Temperature was controlled using a circulating water bath, and sample homogeneity was maintained by magnetic stirring. Samples were equilibrated in the fluorometer sample compartment for 15 min at the initial experimental temperature. Following that initial equilibration, fluorescence data were acquired. The sample was then given 10 min for re-equilibration when temperature was adjusted by single-degree increments or 15 min when the adjustment was more than 5°C. The probe was excited at 540 nm. Emission intensity spectra were measured between 550 and 700 nm. Data were reported either as total intensity (calculated at 585 nm) or as the peak ratio (intensity at 585 nm divided by that at 621 nm). Anisotropy was assayed at 5 nm wavelength increments between 580 and 625 nm using Glan-Thompson polarizers and standard techniques [39]. Interference from scattered light was estimated from vesicle samples without probe and found to be negligible at the wavelengths and conditions used. Spectra shown in the figures are therefore uncorrected for light scatter. Excited-state lifetime values for interpretation of anisotropy results were obtained using an ISS Koala spectrofluorometer in the frequency-modulation domain at the Laboratory for Fluorescence Dynamics (Irvine, CA). A bandpass filter with cutoff at 585 nm and a longpass filter (50% transmission at 600 nm) were used to isolate the blue and red edges of MC540 emission.

### Color phase maps

Maps of membrane phase properties in two dimensions (temperature and cholesterol concentration) were generated from anisotropy, emission intensity, and peak ratio measurements as described previously [40]. Data were normalized and scaled to the value observed at 24-25°C and the extreme value observed at temperatures above 45°C. The normalization was justified based on regression of the raw values of each parameter at these temperature extremes. In each case, the statistical analysis revealed that differences in parameter values at the ends of the temperature profile were due to random rather than systematic effects. Each normalized datum was assigned a color value between 0 and 255 for its respective hue (anisotropy = green, intensity = red, ratio = blue). For intensity and ratio, a normalized value of one was assigned a color value of 255 (brightest) and values less than one were multiplied by 255 to convert them to scaled color intensities. For anisotropy, color values were assigned in a similar fashion with one exception. After initial scaling, the resulting color values were then subtracted from 255 so that color brightness of the green color map followed the same pattern as those for the other two parameters (*i.e. *brighter color = more fluid membrane).

### Cultured Cells

S49 murine lymphoma cells were cultured and prepared for experiments as described [41]. Two ml of washed cells were added to a fluorometer sample cell with 170 nM MC540 and allowed to incubate at 37°C for 5 min. Anisotropy was assayed at 5 nm wavelength increments between 580 and 625 nm. (Emission intensity and peak ratio were also calculated from those data.) Following anisotropy measurements, a calcium ionophore, ionomycin, was added (300 nM) and allowed to incubate with the sample for 10 min. Anisotropy data were then collected again.

## 3. Results

### Steady state anisotropy

**Figure 1:**
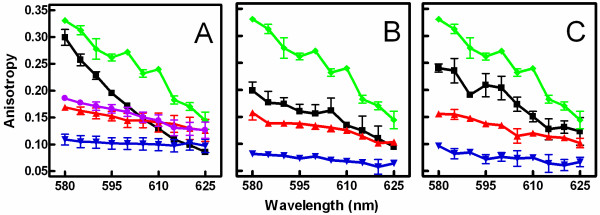
**MC540 steady-state anisotropy spectra at various temperatures**. Panel A: Vesicles were stored at 4°C. Anisotropy was assayed at four temperatures 15 (green), 25 (black), 38 (red), and 46°C (blue). Following collection of anisotropy data at 46°C, the samples were cooled back to 25°C and anisotropy was re-measured (violet). Error bars represent the SEM (n = 3-7) vesicle samples. Panel B: Vesicles were stored at room temperature and anisotropy was assayed at 25 (black), 38 (red), and 46°C (blue). Panel C: The vesicles of Panel B were stored overnight at 4°C after the data in Panel B had been gathered. The anisotropy was then assayed again at 25 (black), 38 (red), and 46°C (blue). Error bars represent the range of two samples in panels B and C. The data at 15°C from Panel A were included in Panels B and C to facilitate comparisons.

In order to achieve the crystalline (L_c_') phase, vesicles were incubated at 4°C for at least 24 h. As shown in Figure 1A, MC540 anisotropy values for vesicles in the L_c_' phase (15°C, green lines and symbols) depended strongly on emission wavelength. The decreasing trend in anisotropy between 580 nm and 625 nm was statistically significant based on linear regression (slope 95% confidence interval = -0.004 to -0.003 nm^-1^, *p *<0.0001, r^2 ^= 0.90). The anisotropy measurements were repeated at 25°C (black), a temperature above the sub-transition (~18°C, Ref. [18]) from the L_c_' to the L_β_' (gel) phase. When this was done, the wavelength dependence persisted, although the overall anisotropy values were lower compared to those at 15°C (slope 95% confidence interval = -0.005 to -0.004 nm^-1^, *p *<0.0001, r^2 ^= 0.91; effects of temperature and wavelength were both significant by two-way analysis of variance (*p *< 0.0001) with significant interaction (*p *= 0.001) that only accounts for 2% of the variance; the temperature effect accounted for 19% of the variance). However, once the sample passed through the pre-transition from the L_β_' to the P_β_' phase (~30°C, [20]), changes across the wavelength spectrum were significantly reduced (38°C, red) and were absent above the main phase transition (46°C, blue; t_m _= ~41.5°C, [22,23]). The slope 95% confidence interval at 38°C = -0.0015 to -0.0004 nm^-1^, *p *= 0.002, r^2 ^= 0.30; at 46°C = -0.0007 to 0.0003 nm^-1^, *p *= 0.4, r^2 ^= 0.01.

One of the well known characteristics of the L_c_' phase is that the kinetics of the transition to and from it are slow (time scale of hours to days, depending on the experimental conditions) such that significant hysteresis is observed as one passes through the sub-transition [19,34,42,43]. We used this phenomenon to determine whether the wavelength dependence at low temperature was due to the L_c_' phase by returning the temperature to 25°C after the vesicles had passed through the main transition. As illustrated by the violet line and symbols in Figure 1A, the wavelength dependence at 25°C following a temperature reversal was greatly attenuated and now indistinguishable from the observations at 38°C (slope 95% confidence interval = -0.002 to -0.001 nm^-1^, *p *< 0.0001, r^2 ^= 0.55). This result indicates that the vesicles were affected by their recent thermal history and suggests that the original data at 25°C represented vestigial effects of the L_c_' phase because of hysteresis through the sub-transition. This interpretation was confirmed by experiments with vesicles that were stored at room temperature continuously since their manufacture (Figure 1B). Similar to the data for vesicles returned to 25°C after passing through high temperature (violet symbols in Figure 1A), the anisotropy trend at 25°C mirrored the trend at 38°C in this experiment. Furthermore, vesicles stored at room temperature and displaying behavior like Figure 1B converted to the pattern of Figure 1A after they were subsequently placed at 4°C for at least 24 h (Figure 1C). This control experiment verified that the results of Figure 1 were reflective of the recent thermal history of the bilayers rather than an artifact of incomplete equilibration during vesicle manufacture.

**Figure 2:**
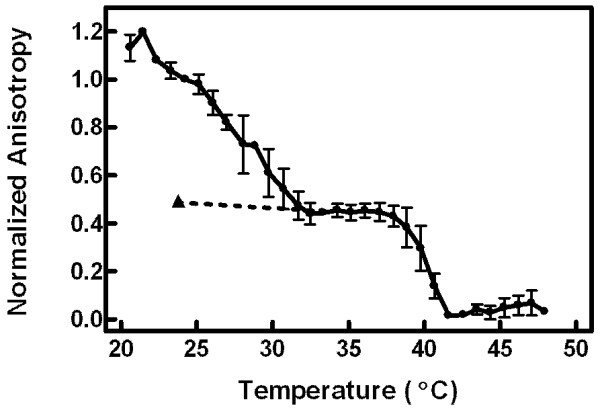
**Detailed anisotropy temperature profile**. Anisotropy was measured at 585 nm in single degree increments from 20 to 48°C. Following the final measurement at 48°C, the sample was cooled to 25°C and anisotropy was re-assessed (triangle, dotted line). The data were normalized to the anisotropy value at 25°C (set to 1.0) and to the minimum value obtain in the profile (set to 0.0) prior to aggregation of results. Error bars represent the SEM for three vesicle samples.

Figure 2 displays a detailed temperature profile of MC540 anisotropy for vesicles stored previously at 4°C ("refrigerated"). The hysteretic recovery of anisotropy from the L_c_' phase was evident for the data from 20 to 32°C. The triangle and dotted line indicate the anisotropy level upon return of the vesicles from high temperature. The main phase transition to the L_α _phase was also apparent in the data at 40-42°C.

### Emission intensity

**Figure 3:**
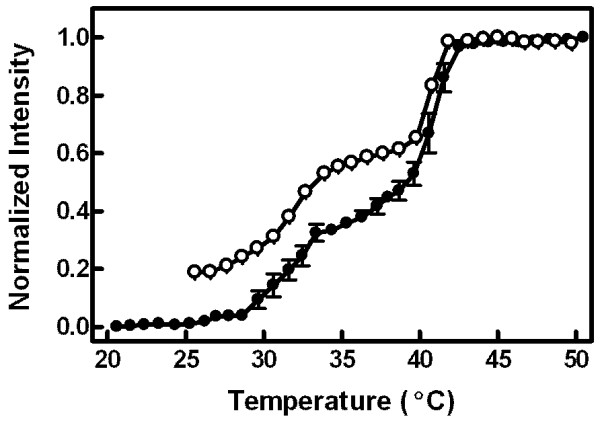
**Detailed emission intensity temperature profile**. Emission intensity (at 585 nm) was collected in single degree increments from 20 to 50°C for vesicles that had been stored at 4°C (solid circles). Error bars represent the SEM for five vesicle samples. The open circles denote a sample that was equilibrated at 50°C prior to cooling the sample to 25°C and then assaying the emission intensity at single degree intervals back up to 50°C. Data were normalized and scaled to the maximum intensity (set to 1.0) and the intensity of refrigerated samples at 25°C (set to 0.0).

Evidence for all four lamellar phases was observed by comparing the temperature profile of emission intensity between refrigerated (Figure 3, solid circles) and equilibrated vesicles (heated to 50°C prior to measurements, open circles). The difference in intensity between 25 and 35°C in these equilibrated vesicles presumably represented the L_β_' and P_β_' phases based on their corresponding temperature ranges [21]. Analogous to the explanation of Figure 2, the additional reduction in intensity observed at low temperatures in the refrigerated vesicles (solid circles, below 29°C) appeared to reflect vestigial effects of the L_c_' phase. The highest intensity in both vesicle preparations corresponded to the L_α _phase.

### Emission spectrum shape

**Figure 4:**
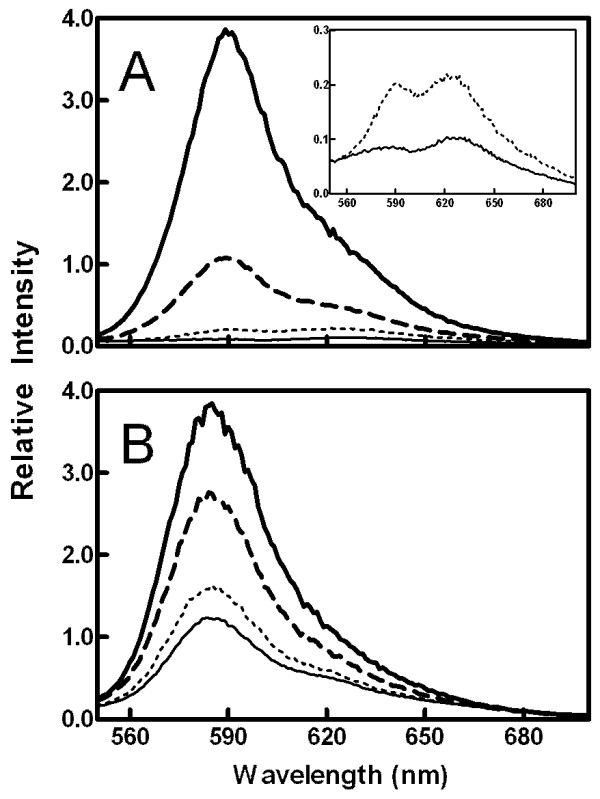
**Emission intensity spectra for refrigerated and equilibrated vesicles**. Emission spectra were collected at 25 (thin solid curve), 30 (dotted), 35 (dashed), and 45°C (heavy solid). Panel A: Vesicles were refrigerated for at least 24 h prior to fluorescence measurements. The inset displays the details of the 25 and 30°C spectra. Panel B: Vesicles were equilibrated at 50°C prior to adjusting to the experimental temperatures used in Panel A.

As shown in Figure 4A, the emission spectrum shape differed between high and low temperatures. The low-temperature spectrum consisted of two peaks (see inset), reflecting separate populations of MC540 in fluorescent monomer (585 nm) and dimer (621 nm) configurations [37]. The single emission peak observed at higher temperatures (dashed and heavy solid curves) presumably reflected monomer fluorescence since prior research has shown that the dimer subpopulation no longer fluoresces at temperatures above the main phase transition [17]. The large increase in monomer fluorescence intensity has been interpreted to represent enhanced probe binding to the membrane and perhaps loss of optical interference and quenching from dimers [13,14,17,44]. Previously, the spectrum showing both monomers and dimers was attributed to the gel phase (L_β_') of the lipids [17]. However, the emission spectrum at 25°C from vesicles previously equilibrated at high temperature mostly lacked the dimer peak, indicating that the membrane properties of the L_c_' phase may contribute more to dimer fluorescence than those of the L_β_' phase (Figure 4B).

**Figure 5:**
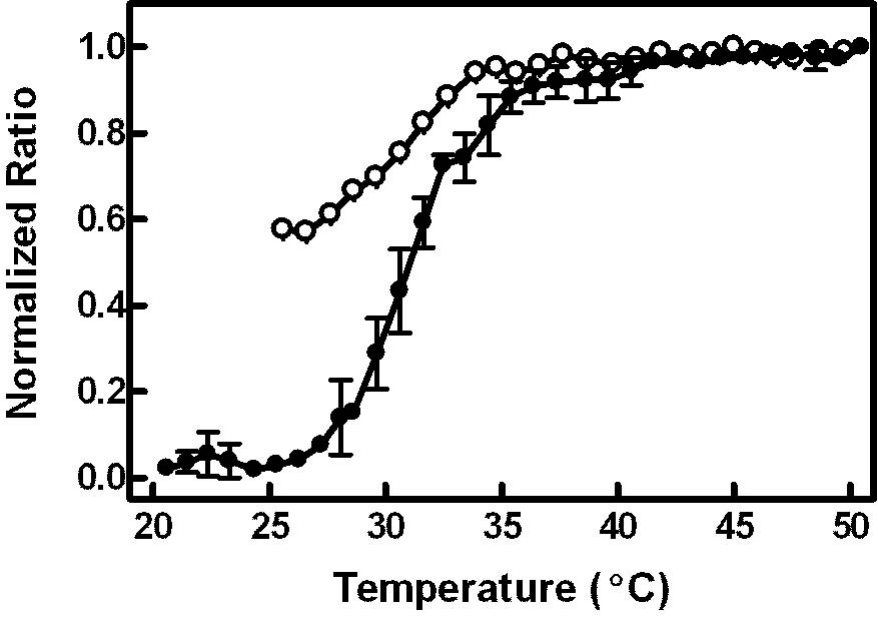
**Detailed emission peak ratio temperature profile**. Emission intensities (at 585 and 621 nm) were collected in single degree increments from 20 to 50°C for refrigerated vesicles (solid circles), and the ratio of these intensities (585 nm/621 nm) was calculated. Error bars represent the SEM for four vesicle samples. The open circles denote a sample that was equilibrated at 50°C prior to cooling the sample to 25°C and then assaying the emission intensity at single degree intervals back up to 50°C. Data were normalized and scaled to the maximum ratio (set to 1.0) and the ratio of refrigerated samples at 25°C (set to 0.0).

Differences in the shape of the spectra were quantified by calculating the ratio of fluorescence intensity at the two spectral peaks (Figure 5). The detailed temperature profiles of the ratios from refrigerated (solid circles) and equilibrated (open circles) vesicles revealed certain similarities to the pair of intensity curves in Figure 3. At temperatures below 28°C, a normalized ratio of 0.6 corresponding to the L_β_' phase was evident in the data from the equilibrated vesicles, and the influence of the L_c_' phase was observed as a much lower ratio (normalized to ~0.05) in the refrigerated vesicles. However, unlike the emission intensity (Figure 3), the ratio of intensities remained constant as the lipids passed through the main transition, indicating no change in the shape of the emission spectra between the P_β_' and L_α _phases (Figure 5).

### Effect of cholesterol

**Figure 6:**
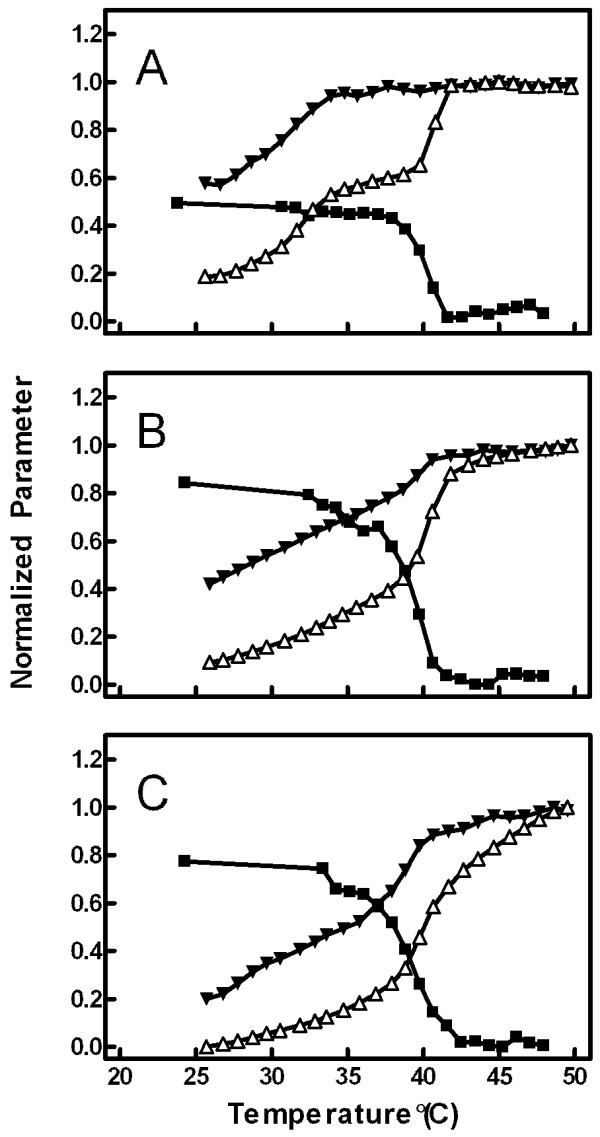
**Effect of cholesterol**. The vesicles were synthesized with 0 mol% (Panel A), 15 mol% (Panel B), or 40 mol% (Panel C) cholesterol. The three measurements-anisotropy (solid squares), emission intensity (open triangles), and peak ratio (solid inverted triangles)-were measured for equilibrated vesicles. Data were normalized as described for Figures 2, 3, and 5.

Figures 6A summarizes the combined observations of MC540 anisotropy (squares), emission intensity (open triangles), and emission peak ratio (solid inverted triangles) for equilibrated DPPC vesicles. To test the effect of the liquid-ordered (L_o_) phase, the experiments were repeated with vesicles containing various concentrations of cholesterol (up to 40%). Cholesterol is known to abolish the pre-transition (thus eliminating the P_β_' phase) and, at concentrations above 25 mol%, establish an L_o _phase that spans the temperature range from about 25 to 50°C [24-26,45]. Consequently, addition of cholesterol altered the temperature trends of anisotropy, intensity, and peak ratio (Panels B: 15% cholesterol, and C: 40% cholesterol). In general, the temperature profiles were smoother as cholesterol content increased, a result consistent with other reports of cholesterol effects on membrane properties [1,26,39,40,46]. Additionally, due to the loss of the P_β_' phase upon addition of cholesterol, the plateaus that existed for all three measurements between about 36 and 39°C were eliminated and replaced by continuous changes in properties reaching an extreme at about 42°C in each case. The main phase transition at ~40-42°C present in the vesicles of pure DPPC was absent at the highest cholesterol concentrations (above 25%) as expected (Figure 6C). Anisotropy in the L_β_' phase was elevated by about 60%. The emission peak ratio and intensity were lower in the L_β_' phase compared to pure DPPC suggesting that monomer fluorescence, and therefore probably monomer binding to the membrane, is decreased by the presence of cholesterol in the solid phase.

**Figure 7:**
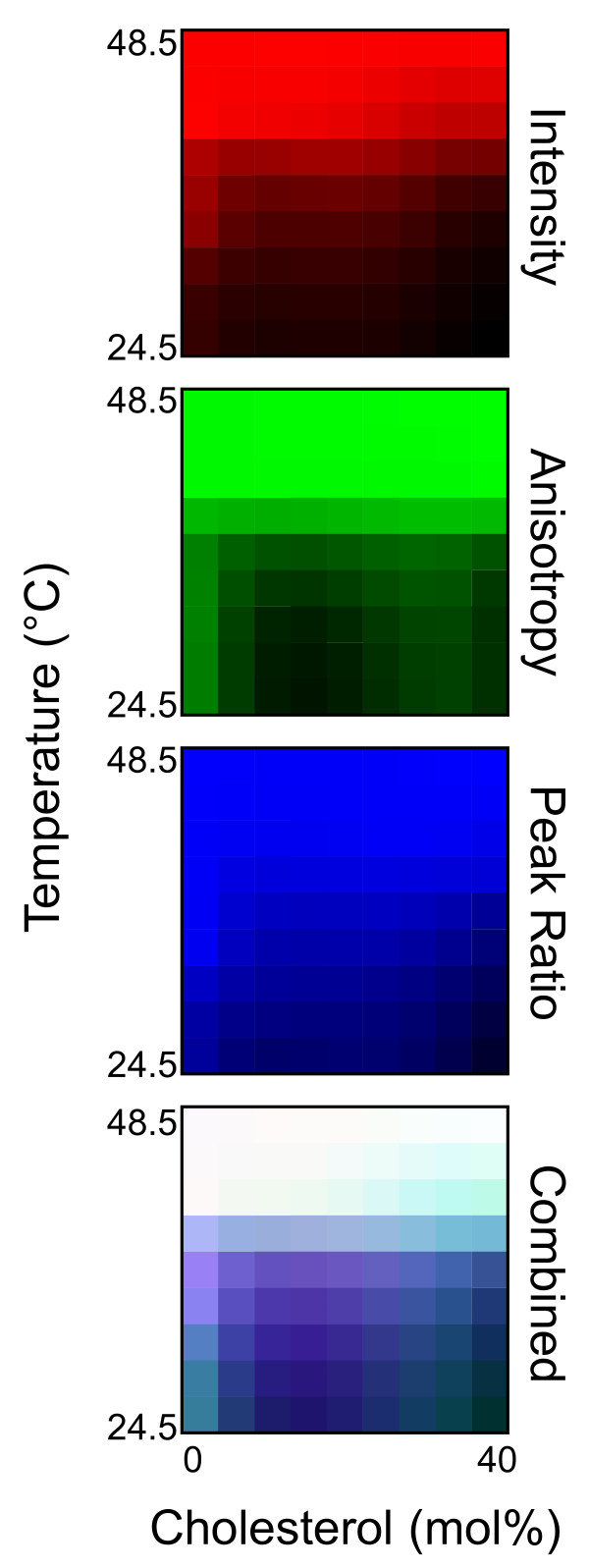
**Temperature and cholesterol phase maps**. The experiments of Figure 6 (equilibrated vesicles) were repeated at multiple cholesterol concentrations between 0 and 40 mol%. Color phase maps were generated at temperature and cholesterol resolution of 3°C and 5 mol% as explained in Materials and Methods. Intensity and ratio maps (red and blue, respectively) were created from the normalized values of the measurements with the brightest color representing a normalized value of 1.0. The anisotropy map (green) was created from normalized anisotropy values with the brightest color representing a normalized value of 0.0. The three maps were then overlaid to create a three-color composite map of the data (bottom panel).

Figure 7 summarizes data from experiments like those shown in Figure 6 as color maps of membrane physical properties detected by the various measurements of MC540 fluorescence. Changes in color brightness indicate differences in parameter values (see legend for details). By overlaying the maps, regions of differing properties relative to cholesterol content and temperature are identified. When the three measurements co-vary, the brightness changes, but the hue remains constant. In contrast, when one or two values change more than the other(s), the hue changes toward the color of the dominant measurement [39,40].

### Application to cultured cells

**Figure 8:**
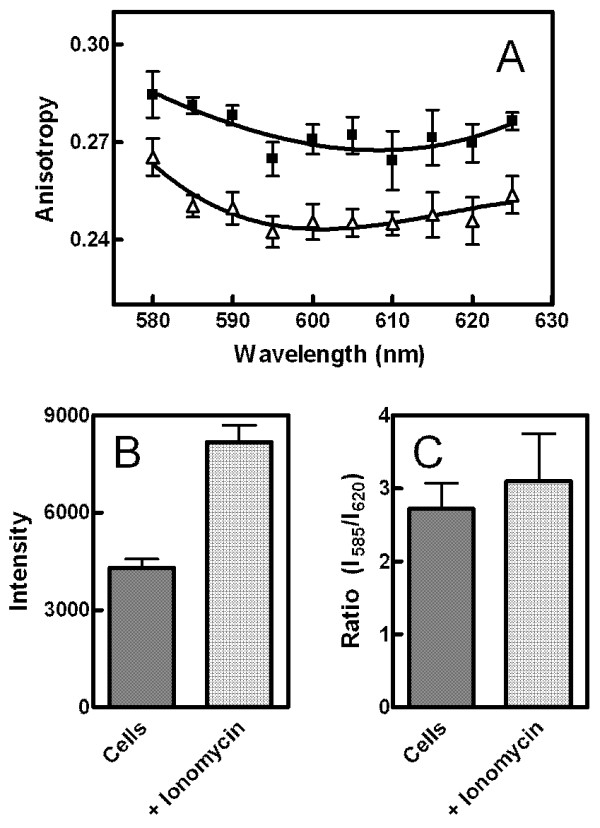
**Application to cultured cells**. Steady-state anisotropy, emission intensity, and peak ratio were assayed for S49 lymphoma cells both prior to and following treatment with ionomycin (300 nM). Panel A: Anisotropy was assayed at multiple wavelengths as described for Figure 1. Both the effects of ionomycin (triangles) and wavelength were statistically significant by two-way analysis of variance (*p *< 0.006 in both cases, n = 9, squares represent the untreated controls). The effect of ionomycin treatment on emission intensity at 585 nm was also significant (Panel B, *p *< 0.0001 by paired t-test, n = 8). The peak ratios (Panel C) were indistinguishable (*p *= 0.33 by paired t-test, n = 8) before and after ionomycin treatment. Error bars represent the SEM.

The three steady-state measurements were repeated with living cells in the presence and absence of a drug (calcium ionophore, ionomycin) known to alter membrane properties [8]. The wavelength dependence of anisotropy was subtle, similar to that observed in Figure 1 for DPPC vesicles above the pre-transition temperature (Figure 8A). Nevertheless, both the effects of wavelength and ionomycin treatment on anisotropy were significant based on two-way analysis of variance (see legend to Figure 8 for details). No significant interaction between the two variables was observed in the analysis of variance (*p *= 0.99), suggesting that ionomycin treatment did not alter the relationship between wavelength and anisotropy. Thus, it appeared that ionomycin, while altering the membrane to allow more motion of MC540, did not change the proportion of fluorescent monomers and dimers. This interpretation was confirmed by an absence of effect of ionomycin on the peak ratio (Figure 8C). As reported previously [8], a significant increase in MC540 fluorescence intensity was observed (Figure 8B).

## 4. Discussion

Evidence of all four lamellar phases was contained in the data in Figures 2, 3, and 5. Recovery from residual effects of the L_c_' phase was evident as a drop in anisotropy (Figure 2) and an increase in both emission intensity (Figure 3) and peak ratio (Figure 5) at temperatures from 20°C up to about 32°C in vesicles that had been stored at 4°C. From 32 to 50°C, the data from these refrigerated vesicles mirrored the results from vesicles that had been equilibrated by storage at room temperature or by raising temperature to 50°C prior to the experiment. The starting values of anisotropy, emission intensity, and peak ratio in vesicles equilibrated at 25°C represented the properties of the L_β_' phase, and the plateau in each between about 35 and 39°C corresponded to the P_β_' phase. Although the properties of the L_β_' and P_β_' phases were distinguishable by both the emission intensity and the peak ratio, measurements of anisotropy revealed identical data for both phases in equilibrated vesicles. Thus, all the changes in anisotropy in refrigerated vesicles up to 32°C were due to vestigial effects of the L_c_' phase. The fluid phase, L_α_, was distinguished from all other phases by high and plateaued emission intensity and minimal anisotropy values (at temperature ≥ 42°C). Interestingly, the peak ratio values did not differentiate between the P_β_' and L_α _phases.

**Figure 9:**
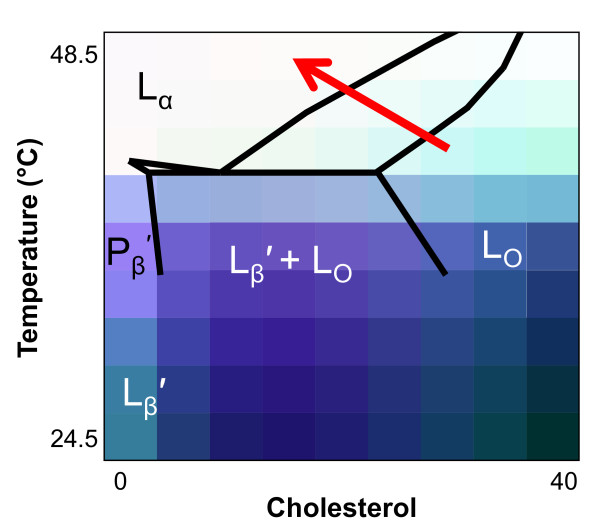
**Interpretations of equilibrated phase map**. A DPPC/cholesterol phase diagram from Ref. [45] was superimposed on the data of the bottom panel of Figure 7. The red arrow approximates the effect of ionomycin on S49 cell membranes in the context of this phase map.

As shown in Figures 6 and 7, the utility of combining three separate measurements from the same probe applies to membranes of mixed composition in addition to pure DPPC. Hence, Figure 7 illustrates resolution of multiple regions of different membrane properties as a function of both temperature and cholesterol concentration. Previous experiments studying the cholesterol phase map with MC540 concluded that the probe behaved as though it were not influenced by the presence of cholesterol [39], which was surprising based on an earlier report of a change in the MC540 emission spectrum upon addition of cholesterol [15]. While superficial inspection of any one of the three observations individually could lead to that interpretation, combining the three demonstrates clearly that the probe can resolve various regions of distinct membrane properties. In fact, these regions matched the boundaries of the traditional cholesterol phase diagram (Figure 9).

One valuable contribution of superimposing multiple measurements of MC540 fluorescence is evident as the heterogeneity of properties (color hues) in the L_o _phase. This heterogeneity has been reported previously from x-ray diffraction studies and interpreted to represent an increase in lipid spacing as temperature is raised in the L_o _phase [47]. However, typical fluorescence observations of the L_o _phase with probes like laurdan and diphenylhexatriene do not detect any significant heterogeneity in that region of the phase diagram [39]. Hence MC540, especially in the context of multiple simultaneous measurements, is able to assess subtleties of bilayer properties at greater resolution than the more conventional steady-state methods with a single probe. An interesting question is whether the combined use of intensity, ratiometric, and anisotropy measurements will also provide insight into the superlattice structures that form at specific cholesterol concentrations [31-33]. The answer is unknown at present since increments of sterol concentration small enough to detect these structures were not used in this study. Nevertheless, since MC540 has been shown to successfully detect superlattice structures [33], it is likely the simultaneous measurements of multiple parameters could reveal additional information about them in future investigations.

Ordinarily, assessment of the anisotropy of fluorescent membrane probes is interpreted as an indication of membrane order and/or "fluidity." In the case of MC540, some of the temperature-dependent changes in anisotropy can also be attributed to the dynamic behavior of MC540 monomer and dimer subpopulations in the membrane. Early studies on the monomer and dimer configurations of MC540 suggested that the mobility of the dimer was greater than that of the monomer when the lipids were in the gel state [16]. These results, together with those from quenching and energy transfer experiments, were interpreted to suggest that the monomers are located near the surface of the membrane, oriented parallel to the phospholipids, and rotationally constrained due to the tight packing of the lipid heads. In contrast, the greater mobility of the dimers was attributed to a perpendicular orientation deep in the membrane [16]. The convincing wavelength dependence of anisotropy observed here at low temperatures (Figure 1) is consistent with those interpretations. Moreover, control experiments assessing differences in probe excited-state lifetimes at long and short wavelengths (as well as high and low temperature) confirmed that the changes in steady-state anisotropy observed in Figures 1 and 2 were explained mostly by differences in probe rotational rates (rather than lifetime). Although these observations and those regarding emission spectra (Figures 1, 2, and 4) corroborate the previous idea that MC540 dimers are fluorescent only in highly packed interfaces [16,17,36], they introduce the novel finding of strong dependence on the L_c_' phase.

Above 32°C, however, anisotropy values are more likely to represent the conventional interpretation of general membrane constraints on probe movement. First, the peak ratio reached a constant value at about 35°C (Figure 5). Inspection of the spectral data (Figure 4) confirms that this constancy is due to an absence of dimer fluorescence at these temperatures. These results suggest that dimer fluorescence vanishes at the pre-transition and clarifies a previous study implying that it is eliminated by the main phase transition [17]. Second, the wavelength dependence of anisotropy observed at 15 and 25°C in refrigerated vesicles was nearly absent at 38°C (Figure 1), suggesting that spectral heterogeneity among MC540 subpopulations no longer is observed above the pre-transition temperature. Thus, additional anisotropy changes at the main transition (Figure 2) must be due to increased rotational freedom as the lipids melt.

The experiments with S49 lymphoma cells indicated that these techniques are applicable to the study of living cells. The modest but reproducible wavelength dependence of anisotropy values suggested that some of the fluorescence observed was due to MC540 dimers (Figure 8A). Although dimer fluorescence would not be expected in a fluid phase, the data of Figure 6C suggested a continuous trend of incremental reduction in dimer fluorescence throughout the L_o _phase. Since at least some of the membranes of cells are expected to have properties analogous to a L_o _phase (lipid rafts) [48], this result of modest wavelength dependence of anisotropy in cells makes sense. Treatment of the cells with ionomycin altered both the anisotropy and emission intensity of MC540 in the samples (Figure 8A, B). However, the wavelength dependence of anisotropy and ratio of peak intensity, *i.e. *both indicating relative proportion of monomer and dimer fluorescence, were not altered (Figures 8A and 8C). Using the color maps created from our data, the relative changes in the membrane following ionomycin treatment were identified; the change was analogous to that observed as DPPC-cholesterol vesicles transition from the L_o _to L_α _phase (Figure 9, red arrow).

The data in this study revealed new insights regarding the properties of lamellar phases in DPPC vesicles and also the behavior of cell membranes during ionophore treatment. First, in addition to confirming previous reports of temperature effects on the L_o _phase, the data shown in Figure 9 suggest a much greater degree of variation across both cholesterol concentration and temperature than previously reported [39,40,47]. Second, as reviewed in [29], the molecular meaning of the mixed phase regions of the phosphatidylcholine/cholesterol phase diagram has been controversial between two disparate models, especially for the region separating the L_α _and L_o _phases: 1) a continuous transition between bordering phases without domains or 2) co-existence of domains (which may or may not necessarily represent equilibrium states) that separately reflect each of the parent phases. These two models are difficult to distinguish with experimental systems such as multilamellar vesicles that cannot be visualized directly by fluorescence microscopy, and evidence supporting both models has been recorded [29]. Interestingly, the data of Figure 9 argue that the situation is different for the transition between L_α _and L_o_, which appears continuous, compared to the transition between L_β_' and L_o_. In the region separating the latter two phases, the figure indicates a combination of physical properties different from either phase suggesting that co-existence of domains can create an environment unique from the parent phases, perhaps analogous to an alloy in metallurgy. Third, although MC540 anisotropy and spectrum peak ratio were stable as a function of temperature in the P_β_' phase of equilibrated vesicles (Figures 2 and 5), results from intensity measurements (Figure 3) revealed gradual changes up to the main phase transition. This observation suggests that the average spacing between phospholipids that allows binding of the probe increases continuously with temperature for DPPC bilayers in the P_β_' phase. Gradual changes were also observed in the L_β_' phase, although some of that result probably represents the breadth of the pre-transition. Fourth, cell plasma membranes are thought to possess characteristics reminiscent of a L_o _phase due to the presence of cholesterol and sphingomyelin-rich domains called rafts (reviewed in [48]). The data in Figure 8 suggest that this trait shifts toward behavior more like a liquid disordered phase when the cells are loaded with calcium as illustrated in Figure 9. This outcome has significance for helping interpret studies designed to assess how the cell membrane changes during programmed cell death [3,4,8].

## 5. Conclusions

In summary, the observations in this study validate our initial postulate and demonstrate the utility of multiple simultaneous steady-state measurements of MC540 fluorescence for discerning lipid phase properties with a single probe. Moreover, we have provided examples of how such measurements can provide new information regarding characteristics these phases in model and biological membranes. Previous studies with other fluorescent probes have allowed detection of more than one phase transition in membranes [1,34,39,40]. However, to our knowledge, this is the first example where five general lamellar phosphatidylcholine phases (L_c_', L_β_', P_β_', L_α_, and L_o_) as well as multiple characteristics within those phases could be detected with a single probe using combined steady-state fluorescence measurements.
